# Vacancy Augmented Piezo‐Sonosensitizer for Cancer Therapy

**DOI:** 10.1002/advs.202301152

**Published:** 2023-07-03

**Authors:** Qingyuan Wu, Jie Zhang, Xueting Pan, Zhijun Huang, Haoyuan Zhang, Juan Guo, Yun Xue, Rui Shi, Huiyu Liu

**Affiliations:** ^1^ Beijing Advanced Innovation Center for Soft Matter Science and Engineering State Key Laboratory of Organic‐Inorganic Composites Beijing Laboratory of Biomedical Materials Bionanomaterials & Translational Engineering Laboratory Beijing Key Laboratory of Bioprocess Beijing University of Chemical Technology Beijing 100029 P. R. China; ^2^ Beijing National Laboratory of Molecular Sciences, Beijing National Laboratory of Molecular Sciences Institute of Chemistry, Chinese Academy of Sciences Beijing 100190 P. R. China; ^3^ National Center for Orthopaedics Beijing Research Institute of Traumatology and Orthopaedics Beijing Jishuitan Hospital Beijing 100035 P. R. China

**Keywords:** molybdenum disulfide, piezoelectricity, sonodynamic therapy, ultrasound, vacancy

## Abstract

Sonodynamic therapy (SDT) has been widely reported as a noninvasive and high‐penetration therapy for cancer; however, the design of an efficient sonosensitizer remains an urgent need. To address this issue, molybdenum disulfide nanoflowers (MoS_2_ NF) as piezo‐sonosensitizers and introduced sulfur vacancies on the MoS_2_ NF (Sv‐MoS_2_ NF) to improve their piezoelectric property for cancer therapy are designed. Under ultrasonic mechanical stress, the Sv‐MoS_2_ NF resulted in piezoelectric polarization and band tilting, which enhanced the charge carrier separation and migration. This resulted in an improved catalytic reaction for reactive oxygen species (ROS) production, ultimately enhancing the SDT performance. Thanks to the high efficiency of ROS generation, the Sv‐MoS_2_ NF have demonstrated a good anticancer effect in vitro and in vivo. Following a systematic evaluation, Sv‐MoS_2_ NF also demonstrated good biocompatibility. This novel piezo‐sonosensitizer and vacancy engineering strategy provides a promising new approach for achieving efficient SDT.

## Graphical abstract

This work describes a strategy that vacancy augments the piezoelectricity of piezo‐sonosensitizer for sonodynamic therapy. Sulfur vacancies are constructed on the molybdenum disulfide by hydrogen peroxide etching to improve the piezoelectric polarization, charge carrier dynamic, and reactive oxygen species generation efficiency of molybdenum disulfide. What's more, the sulfur vacancies are also helpful for increasing tumor inhibition. This novel vacancy engineering strategy provides a promising new approach for achieving efficient sonodynamic therapy.

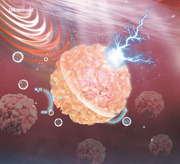

## 1 Introduction

Sonodynamic therapy (SDT) has recently emerged as a promising cancer treatment due to its noninvasive nature and ability to penetrate deeply into tissues.^[^ ^1^ ^]^ The efficacy of SDT largely depends on the performance of sonosensitizers, which are activated by ultrasound (US) to generate reactive oxygen species (ROS). However, conventional inorganic sonosensitizers, such as titanium dioxide,^[^ ^2^ ^]^ exhibit limited ROS production yield owing to rapid charge carrier recombination. Therefore, there is a pressing need to develop novel sonosensitizers with enhanced carrier dynamics for improved ROS generation. Various strategies, including heteroatom doping and heterojunction construction, have been employed to improve carrier separation.^[^ ^3^ ^]^ Nevertheless, regulating the doping concentration within an optimal range remains challenging. Low doping level fails to yield significant improvements in catalysts performance,^[^ ^4^ ^]^ while excessive doping concentrations inadvertently create recombination sites for charge carriers. Although composite materials can form heterojunctions that generate a built‐in electric field to promote effective carrier separation, these static fields are readily shielded by the continuous generation of charge carriers, leading to unsustainable separation. In contrast, piezoelectric materials are capable of generating built‐in electric fields via polarization under mechanical forces, such as US.^[^ ^5^ ^]^ Consequently, piezoelectric materials hold considerable promise as sonosensitizers, enabling sustained improvements in carrier separation and facilitating efficient ROS production.

Two‐dimensional semiconductors like molybdenum disulfides (MoS_2_) possess the advantage of being easily deformed and having high strain toleration, rendering them ideal candidates for piezoelectric materials.^[^ ^6^ ^]^ MoS_2_, is believed to demonstrate piezoelectric property based on its noncentrosymmetric structure.^[^ ^7^ ^]^ Particularly, MoS_2_ with few layers or single layer exhibits strong piezoelectric responses.^[^ ^8^ ^]^ Furthermore, MoS_2_ has excellent biocompatibility and has been widely applied in biological applications such as photothermal therapy, nanozymes, immunotherapy, and so on.^[^ ^9^ ^]^ Therefore, MoS_2_ has the potential to be a safe and effective piezo‐sonosensitizer. However, the MoS_2_ plane itself is catalytically inert, and only the dangling bongs of Mo and S at the edge of MoS_2_ can adsorb water or oxygen to generate reactive oxygen species (ROS), which limits its catalytic efficiency.^[^ ^10^ ^]^ Hence, it is necessary to construct active sites on the MoS_2_ plane to modulate their piezoelectric properties.

**Scheme 1:**
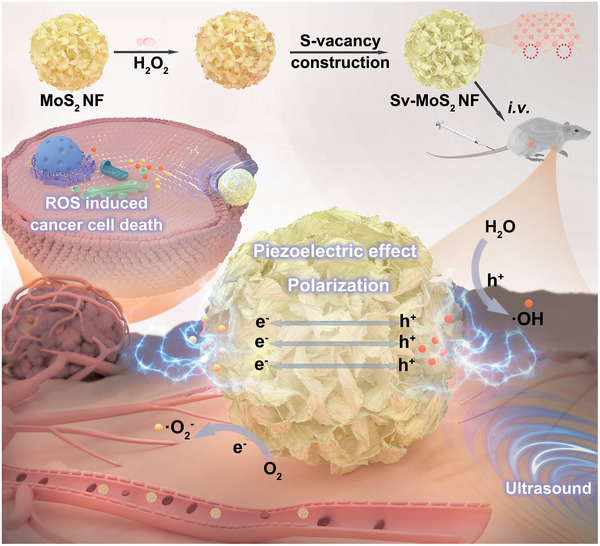
Schematic illustration of vacancy‐modulated piezoelectric sonodynamic therapy.

In this study, we aimed to engineer the piezoelectric properties of MoS_2_ to improve the SDT effect by creating sulfur (S)‐vacancy by etching (**Scheme** **1**). Importantly, to the best of our knowledge, this is the first study wherein defect engineering was applied to modulate the piezoelectricity of piezo‐sonosensitizer. MoS_2_ nanoflowers (MoS_2_ NF) were synthesized successfully using a hydrothermal method and S‐vacancies were constructed by etching with H_2_O_2_ (hereafter abbreviated as Sv‐MoS_2_ NF). The Sv‐MoS_2_ NF demonstrated high SDT performance, which may be attributed to the improved piezoelectric property to increase charge carrier separation and ROS generation. Both in vitro and in vivo experiments revealed that the Sv‐MoS_2_ NF exhibited good biocompatibility and effective SDT efficacy for tumor ablation, which shows a 85.4% tumor inhibition rate. Our findings highlight that the vacancy construction strategy is beneficial for improving the SDT effect of piezo‐sonosensitizers and provides a novel nanoplatform for piezo‐sonosensitizers design.

## 2 Result and Discussion

### 2.1 Preparation and Characterization of Sv‐MoS_2_ NF

**Figure 1:**
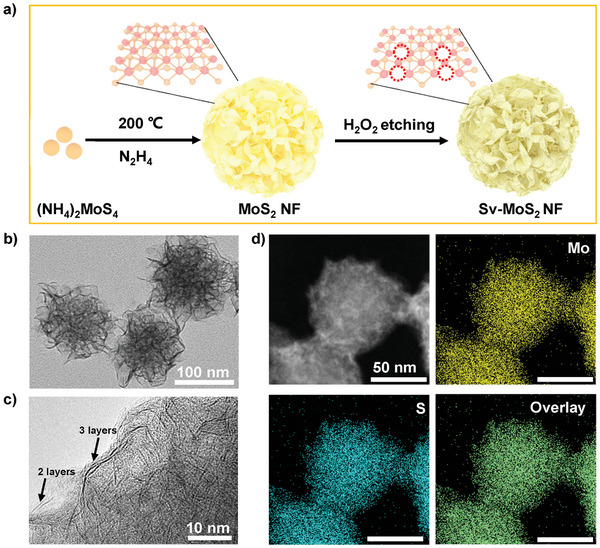
The synthesis and characterization of MoS_2_ NF. a) Schematic diagram of the MoS_2_ NF synthesis. b) TEM image of MoS_2_ NF. c) High‐resolution TEM image of MoS_2_ NF. d) Element‐mapping images of MoS_2_ NF.

In this work, MoS_2_ NF were synthesized by a one‐pot hydrothermal method using ammonium tetrathiomolybdate ((NH_4_)_2_MoS_4_) as both Mo and S precursors (**Figure** **1**a).^[^ ^11^ ^]^ In Figure 1b, the morphology and size of MoS_2_ NF were measured by transmission electron microscope (TEM). The image shows that MoS_2_ NF with sizes ≈110 nm are composed of numerous nanosheets. The high‐resolution TEM reveals that each petal of MoS_2_ NF consists of few‐layered MoS_2_ nanosheets (Figure 1c). Excitingly, the few‐layered MoS_2_ nanosheets potentially tend to show high piezoelectric properties.^[^ ^7b^ ^]^ In this work, the crystal structure of MoS_2_ contained octahedral (1T) phase, and trigonal prismatic (2H) phase. Raman features of 2H and 1T phase MoS_2_ were displayed in Figure S1a (Supporting Information). The characteristic peaks at 150, 238, and 339 cm^−1^ were denoted as J_1_, J_2_, and J_3_, respectively, which are attributed to the zone‐folding mechanism acting in 1T phase but not allowing in 2H phase MoS_2_. The characteristic peaks at 287, 377, and 404 cm^−1^ arose from E_1g_, E^1^_2g_, and A_1g_.^[^ ^12^ ^]^ Homogeneous distribution of Mo and S can be found in the elemental mapping images (Figure 1d). The X‐ray diffraction (XRD) result indicates that the peaks at 14.1°, 33.4°, and 57.8° belong to the (002), (100), and (110) planes of MoS_2_, respectively (Figure S2, Supporting Information). X‐ray photoelectron spectroscopy (XPS) investigates the chemical bonding information of MoS_2_ NF (Figure S3, Supporting Information).^[^ ^13^ ^]^ The peaks at 228.5 eV and 231.9 eV in the Mo 3d spectrum belong to the binding energies of Mo^4+^ 3d_5/2_ and 3d_3/2_ of 1T phase MoS_2_, respectively. A new doublet of Mo 3d spectrum located at 229.7 eV and 233.3 eV indicates the production of 2H MoS_2_ and the peak at 225.8 eV belongs to S^2−^ 2s. The peak at 231.3 eV and 235.0 eV are corresponding to Mo^5+^ 3d_5/2_ and 3d_3/2_, respectively. The S 2p spectrum shows peaks at 161.3 eV and 162.8 eV corresponding to S 2p_3/2_ and S 2p_1/2_, respectively. The S 2p spectrum also shows a new doublet at 162.3 eV and 164.1 eV, which is assigned to the characteristic peaks of distorted 1T phase MoS_2_. After the MoS_2_ NF is synthesized, the H_2_O_2_ chemical etching strategy is carried out to improve S‐vacancies on the MoS_2_ NF.^[^ ^14^ ^]^ The MoS_2_ NF were immersed into H_2_O_2_ for different duration times (10, 30, 50, 70, and 90 min) and named M10, M30, Sv‐MoS_2_ NF, M70, and M90, respectively. The TEM images of them were shown in Figure S4 (Supporting Information). Dynamic light scattering (DLS) results indicate that their hydrated‐particulate sizes have no significant changes after H_2_O_2_ etching (Figure S5, Supporting Information). While, the zeta potentials of them change significantly after H_2_O_2_ is etched (Figure S6, Supporting Information). Additionally, XPS analyzes the changes in elemental chemical states and surface S‐vacancy concentration. The S‐vacancy concentration can be calculated by the S:Mo ratio of the signals of the Mo 3d and the S 2p regions. The S‐vacancy concentration increase with the etching time prolonged and reach the maximum after 50 min of H_2_O_2_ etching (Figure S7, Supporting Information). In the Figure S1b (Supporting Information), comparing with MoS_2_ NF, the slight shift (∼2 cm^−1^) of the E^1^_2g_, and A_1g_ peaks of Sv‐MoS_2_ NF was attributed to the decreasing number of Mo–S bonds accompanied by the increased amount of in‐plane S‐vacancies.^[^ ^15^ ^]^ The results mean that the H_2_O_2_ chemical etching strategy successfully constructs more S‐vacancies on the surface of MoS_2_ NF. The H_2_O_2_ chemical etching strategy also induces the value states change of Mo element. In the high‐resolution XPS, the Mo^5+^:Mo^4+^ rate was 13.49% before H_2_O_2_ treatment, which increased to 16.93% after H_2_O_2_ treatment with 50 min (Figures S3 and S8, Supporting Information). The S‐vacancy was also detected by an electron spin resonance spectrometer (ESR). The characteristic peak of the Mo‐S dangling bonds can be detected ≈3500 G (Figure S9, Supporting Information).^[^ ^16^ ^]^ The dangling bonds originate from the presence of S‐vacancies in the MoS_2_ NF. The multiple splitting of the peak ascribes to the signals of the 1T phase incorporate with the 2H phase.^[^ ^12^ ^]^ The S‐vacancies signal at 3620 and 3512 G belong to the 1T phase and 2H phase, respectively. The multiple peaks ≈3400 to 3300 G are related to the signal of Mo^5+^.^[^ ^17^ ^]^ The peak at 3121 may ascribe to the conduction electrons.^[^ ^18^ ^]^ Initially, the signals of S‐vacancy increased with the etching duration increase until it reaches 50 min. After that, the signals decreased with the etching duration increase. This phenomenon is consistent with the result of the XPS test.

### 2.2 Piezoelectric Properties and Sonocatalytic Effect of Sv‐MoS_2_ NF

**Figure 2:**
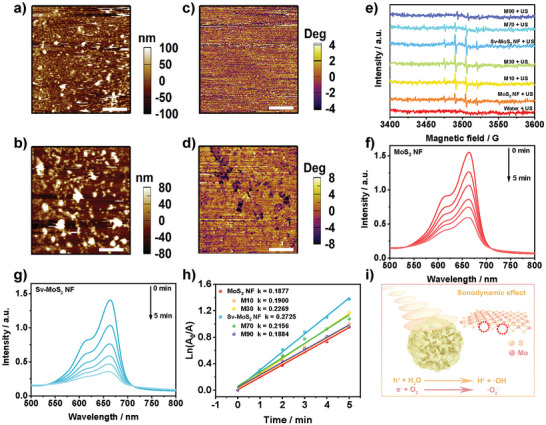
Sonodynamic and piezoelectric performance detection. AFM images of a) MoS_2_ NF and b) Sv‐MoS_2_ NF. PFM phase images of c) MoS_2_ NF and d) Sv‐MoS_2_ NF. Scale bars = 5 µm. e) ESR spectra of •OH generated by MoS_2_ NF and etched MoS_2_ under US irradiation (1.0 MHz, 1.5 W cm^−2^, 1 min). Time‐dependent sono‐degradation of MB indicating •OH generated by f) MoS_2_ NF, g) Sv‐MoS_2_ NF under US irradiation (1.0 MHz, 1.5 W cm^−2^). h) Rates constant for MB decomposition. i) Schematic diagram of the ROS generation under US irradiation.

The piezoelectric properties of MoS_2_ have been confirmed by piezoelectric force microscopy (PFM). The atom force microscopy (AFM) images and the corresponding PFM phase images reveal the irregularly shaped domain walls (**Figure** **2**a‐d and Figure S10, Supporting Information). PFM was also used to verify the local polarization switch of MoS_2_. A typical butterfly amplitude curve and phase chart between a ramp voltage from −5 to +5 V was measured (Figure S11, Supporting Information). The estimated piezoelectric constant based on the amplitude‐voltage butterfly loops of Sv‐MoS_2_ NF is 270 pm V^−1^, which is higher than 165 pm V^−1^ of MoS_2_ NF. It means that the H_2_O_2_ etching strategy can improve the piezoelectric property of MoS_2_ by constructing more S‐vacancies. The increase in S‐vacancies may potentially lead to the increased center asymmetry in Sv‐MoS_2_ NF, which needs further experiments to be verified. Subsequently, ESR measurement was carried out with 5,5‐dimethyl‐1‐pyrroline N‐oxide (DMPO) as a trapping agent hydroxyl radical (•OH) and superoxide anion (•O_2_^−^),^[^ ^19^ ^]^ to evaluate the sonocatalytic efficiency of MoS_2_ NF and the series of etched MoS_2_ (including M10, M30, Sv‐MoS_2_ NF, M70, and M90). The typical 1:2:2:1 quartet signal is attributed to a DMPO spin adduct of •OH. The spectra show that the etched MoS_2_ NF possess a higher •OH level than MoS_2_ NF under US irradiation (Figure 2e and Figure S12, Supporting Information). What's more, Sv‐MoS_2_ NF + US group has the highest •OH generation rate above other groups and increased by 43.4% than the MoS_2_ NF + US group. The ESR spectra of MoS_2_ also show the •O_2_^−^ generation under US irradiation (Figure S13, Supporting Information). Additionally, •OH generation was also detected by methylene blue (MB) degradation evaluation (Figure 2f‐g and Figure S14, Supporting Information). The characteristic peak at 665 nm decreased with the increase of US irradiation time. As shown in Figure 2h, the degradation rate constant of the Sv‐MoS_2_ NF + US group is 0.2725 min^−1^, which is higher than the MoS_2_ NF group (0.1877 min^−1^) by 45.2%. These results imply that the S‐vacancy concentration of MoS_2_ can significantly improve the piezoelectric property and then enhance the sonocatalytic effect of MoS_2_ to generate more ROS (Figure 2i).

### 2.3 Sonocatalytic Mechanism of Sv‐MoS_2_ NF

**Figure 3:**
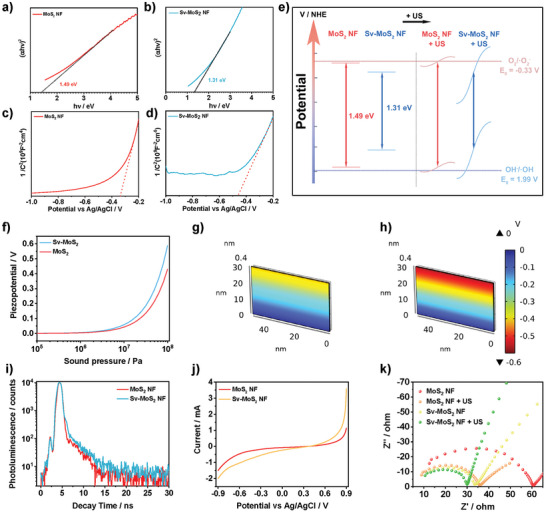
Sonodynamic mechanism of MoS_2_. Band gaps of a) MoS_2_ NF and b) Sv‐MoS_2_ NF. Mott‐Schottky plots of c) MoS_2_ NF and d) Sv‐MoS_2_ NF. e) Schematic illustration of band tilting under applied strain by US‐driven pressure and the accompanied redox reaction. f) The piezopotential of MoS_2_ NF and Sv‐MoS_2_ NF under varied pressure simulated by COMSOL. The piezopotential distribution in g) MoS_2_ NF and h) Sv‐MoS_2_ NF simulated by COMSOL. i) The decay time detected by time‐resolved photoluminescence spectroscopy. j) Linear sweep voltammograms of MoS_2_ NF and Sv‐MoS_2_ NF. k) EIS of MoS_2_ NF and Sv‐MoS_2_ NF with or without US irradiation (1.0 MHz, 1.5 W cm^−2^).

To clarify the potential mechanism between the piezoelectricity and sonocatalytic effect, experiments were carried out to evaluate the process of charge separation and transfer. Initially, the band structure was measured. The band gaps of MoS_2_ NF were calculated by the plotted Tauc plot of the Kubelka–Munk function. Obviously, the band gaps of etched MoS_2_ NF are narrower than that of MoS_2_ NF (**Figure** **3**a‐b and Figure S15, Supporting Information). It is related to the fact that S‐vacancy changes the distribution of 4d orbital electrons and changes the formation of a donor‐like level.^[^ ^15^ ^]^ The flat band potentials were measured and calculated from the Mott–Schottky curve. The Mott–Schottky plots with positive slopes investigate that all of the MoS_2_ NF are typical n‐type semiconductors (Figure 3c‐d and Figure S16, Supporting Information). The flat band potential measured by the Mott‐Schottky method is higher than conduction bands (CB) by ≈0.1 V in n‐type semiconductors. Then, the calculated CB are 0.30, 0.28, 0.23, 0.18, 0.20, and 0.24 V of MoS_2_ NF, M10, M30, Sv‐MoS_2_ NF, M70, and M90, respectively. According to the results of the band gap and CB, the valance band (VB) can be calculated as 1.79, 1.67, 1.62, 1.49, 1.55, and 1.64 V, which belong to MoS_2_ NF, M10, M30, Sv‐MoS_2_ NF, M70, and M90, respectively. In fact, the electrons generated at the bottom of the CB of MoS_2_ NF are energetically unfavorable for •O_2_^−^ generation (the redox potentials of O_2_/•O_2_^−^ is −0.33 V), and the holes generated at the top of the VB of MoS_2_ NF are also energetically unfavorable for •OH generation (the redox potentials of H_2_O/•OH is −1.99 V) (Figure 3e). Considering the MoS_2_ NF and Sv‐MoS_2_ NF with a broad range absorption from ultraviolet ray to near‐infrared light (NIR), a photocatalytic experiment of MoS_2_ NF and Sv‐MoS_2_ NF was applied for MB decomposition to detect the •OH generation (Figure S17, Supporting Information). The degradation rates constant of the MoS_2_ NF group (0.0174 min^−1^) and Sv‐MoS_2_ NF group (0.0210 min^−1^) are higher than the water group (0.0012 min^−1^), which can be attributed to the dye absorption. The degradation rate constants of the MoS_2_ NF + NIR light group and Sv‐MoS_2_ NF + NIR light group are 0.0172 min^−1^ and 0.0240 min^−1^, respectively. They are no significant differences with or without light irradiation, which verify the energetically unfavorable •OH generation for MoS_2_ NF and Sv‐MoS_2_ NF (Figure S18, Supporting Information). Therefore, the degradation of MB under US irradiation is related to the tilted VB by the piezoelectric internal electric field. The piezopotential modulates the VB and makes it energetically favorable for •OH generation. According to the piezoelectric constant, the piezopotential can be estimated by equation, and the detail can be seen in supporting information. The piezopotential also can be numerically simulated by COMSOL multiphysics software based on the measured piezoelectric constants (Figure 3f‐h). It reveals that the piezopotential is positively related to sound pressure. The simulated piezopotentials are 0.59 V of Sv‐MoS_2_ NF and 0.43 V of MoS_2_ NF, which are similar to the calculation of the equation.

Considering that Sv‐MoS_2_ NF has the highest S‐vacancy concentration above other samples, it was compared with MoS_2_ NF to evaluate the charge carrier dynamic modulated by vacancy. Time‐resolved transient photoluminescence (TRPL) spectra showed the lifetimes of electron‐hole pairs for MoS_2_ NF and Sv‐MoS_2_ NF (Figure 3i). The fluorescence lifetime curves were curve‐fitted using double exponential decay kinetics:

(1) $$I\left(t\right)=B_{1}e^{-t\;/\tau_{1}}+B_{2}e^{-t\;/\tau_{2}}$$

(2) $$\tau_{\mathrm{ave}}=\;\frac{B_{1}\tau_{1}^{2}+\;B_{2}\tau_{2}^{2}}{B_{1}\tau_{1}+\;B_{2}\tau_{2}}$$

In the equation, (𝑡): the fluorescence intensity. *τ*_1_: the fast decay time, *τ*_2_: the slow decay time, *τ*_ave_: the average decay time, B_1_: the weighting factor of *τ*_1_, B_2_: the weighting factor of *τ*_2_. The fitted parameters can be seen in Table S1 (Supporting Information). Here, *τ*_1_ is related to the nonradiative recombination of the electrons with the surface vacancies. *τ*_2_ is related to the free excitons interband recombination.^[^ ^20^ ^]^ Both the average decay time and fast decay time of Sv‐MoS_2_ NF are longer than that of MoS_2_ NF, which indicated that the modulating of S‐vacancy facilitated interfacial charge manipulation then improving the charge separation. The charge carrier dynamic separation process was also conducted by linear sweep voltammograms (LSV). When the potential was applied, Sv‐MoS_2_ NF showed a higher current density than MoS_2_ NF, demonstrating the migration of more generated charges in Sv‐MoS_2_ NF (Figure 3j). Besides, the electrochemical impedance spectra (EIS) were performed to analyze the separation efficiency of carriers. The arc radius in the Nyquist plot represents the transfer resistance of charges (Figure 3k). The small arc radius of Sv‐MoS_2_ NF reveals a low resistance for charge transfer. Surprisingly, both the arc radius of Sv‐MoS_2_ NF and MoS_2_ NF decreased under US irradiation, inferring that piezoelectric polarization strongly affects the charge carrier separation process. Thus, effective electron‐hole pair separation and fast charge transfer can be modulated by S‐vacancy in the MoS_2_ NF under US irradiation then improve the ROS generation efficiency.

### 2.4 In Vitro Sonodynamic Anticancer Effect of Sv‐MoS_2_ NF

**Figure 4:**
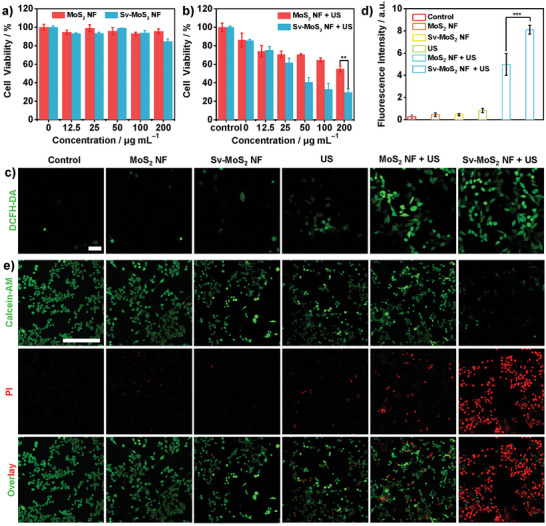
In vitro SDT of MoS_2_ NF and Sv‐MoS_2_ NF for 4T1 cells. a) Relative cell viability of 4T1 cells after treating with MoS_2_ NF and Sv‐MoS_2_ NF. b) Relative cell viability of 4T1 cells after different treatments. c) CLSM images and d) the fluorescence quantification of 4T1 cells stained with DCFH‐DA after different treatments. e) CLSM images of 4T1 cells after different treatments, which were stained by PI (red, dead cells) and Calcein‐AM (green, live cells). Scale bar = 250 µm. *P < 0.05, **P < 0.01, ***P < 0.001.

To explore the therapeutic efficiency of MoS_2_ NF and Sv‐MoS_2_ NF under US irradiation, a standard methyl thiazolyl tetrazolium (MTT) assay protocol was carried out to evaluate the in vitro cytotoxicity. Different concentrations of MoS_2_ NF and Sv‐MoS_2_ NF were incubated with 4T1 breast cancer cells. No significant viability change was found in the concentration range from 0 to 200 µg mL^−1^ (**Figure** **4**a). Then, different concentrations of MoS_2_ NF and Sv‐MoS_2_ NF were incubated with 4T1 cells for 24 h and followed by US irradiation (1.0 MHz, 1.5 W cm^−2^, 3 min). The results of the MTT assay show that the cell viabilities of MoS_2_ NF and Sv‐MoS_2_ NF under the concentrations of 200 µg mL^−1^ are 55.0% and 29.4%, respectively (Figure 4b). The SDT effect of the Sv‐MoS_2_ NF group is 46.5% higher than the MoS_2_ NF group. Afterward, 2,7‐dichlorofluorescein diacetate (DCFH‐DA) was carried out to detect the ROS generated at the cellular level. DCFH‐DA can be hydrolyzed as 2,7‐dichlorofluorescein (DCFH) by intracellular esterase. Then, 2’,7’‐dichlorofluorescein (DCF) with green fluorescence is derived from DCFH oxidized by ROS. In Figure 4c, after Sv‐MoS_2_ NF and MoS_2_ NF were irradiated by the US, a strong green fluorescence can be observed through confocal laser scanning microscopy (CLSM), indicating effective ROS generation during the US irradiation. The fluorescence intensities of the Control group, MoS_2_ NF group, Sv‐MoS_2_ NF group, US group, MoS_2_ NF + US group, and Sv‐MoS_2_ NF + US group were counted as 0.26, 0.45, 0.45, 0.83, 4.97, and 8.10, respectively. The Sv‐MoS_2_ NF + US group is 38.4% higher than the MoS_2_ NF + US group (Figure 4d). In other words, Sv‐MoS_2_ NF has higher sonodynamic efficiency than MoS_2_ NF, which is consistent with the results of the ESR experiment. The therapeutic effect was also evaluated by CLSM. Calcein acetoxymethyl ester (Calcein‐AM) and propidium iodide (PI) were applied for staining live cells (green) and dead cells (red), respectively (Figure 4e). The results showed that almost all of the cells were killed in the Sv‐MoS_2_ NF + US group and a part of the cells was killed in the MoS_2_ NF + US group, which indicated that both MoS_2_ NF and Sv‐MoS_2_ NF possess strong SDT efficiency under US irradiation.

### 2.5 In Vivo Sonodynamic Anticancer Effect of Sv‐MoS_2_ NF

**Figure 5:**
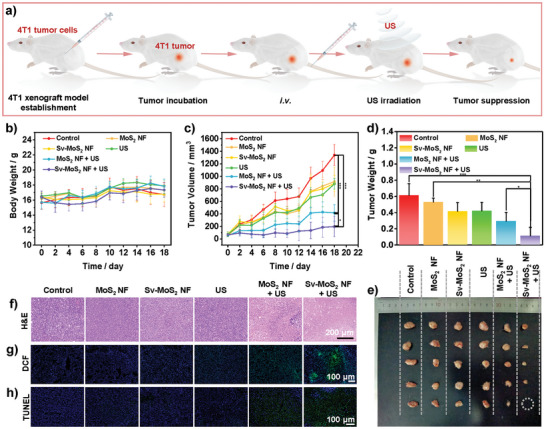
In vivo therapy of 4T1 breast tumor‐bearing mice. a) Illustration of the establishment of breast tumor model. b) Body weight and c) tumor volume of different groups during the 18‐days therapeutic period. d) Tumor weight and e) tumor photograph of different groups on the 18th day. f) H&E, g) DCF, and h) TUNEL stain assay for tumor after different treatments. *P < 0.05, **P < 0.01, ***P < 0.001. US irradiation: 1.0 MHz, 1.5 W cm^−2^, 50% duty cycle, 5 min.

Considering the good results of in vitro experiments, in vivo therapeutic effect of Sv‐MoS_2_ NF and MoS_2_ NF as sonosensitizers were evaluated. Initially, photoacoustic (PA) imaging was used for measuring the accumulation of Sv‐MoS_2_ NF and MoS_2_ NF in the mice bearing 4T1 tumor (Figure S19, Supporting Information). At the 18th hour after the tail intravenous injection (*i.v*.), strong PA signals can be observed from Sv‐MoS_2_ NF and MoS_2_ NF, respectively. It indicated that Sv‐MoS_2_ NF and MoS_2_ NF can accumulate in the tumor by passive tumor targeting. Then, six groups (Control, MoS_2_ NF, Sv‐MoS_2_ NF, US, MoS_2_ NF + US, and Sv‐MoS_2_ NF + US) were divided to assess the therapeutic efficiency for the mice bearing 4T1 tumor. The establishment of 4T1 xenograft tumor model in mice and the antitumor experiments were shown in **Figure** **5**a. The irradiation time point of US was guided by the results of PA imaging. To evaluate the curative effect, the tumor volume and body weight of mice were recorded at different time points (0, 2,4, 6, 8, 10, 12, 14, 16, and 18 days). Figure 5b shows that the body weight of the mice in all groups gets a slight increase and no significant difference can be seen between Sv‐MoS_2_ NF and other groups. The tumor volume data in Figure 5c exhibits significant tumor suppression of the MoS_2_ NF + US, and Sv‐MoS_2_ NF + US groups, whose tumor inhibition rates are 69.0% and 85.4%, respectively. The mice were sacrificed on the 18th day, then the tumors were collected, weighed, and photographed (Figure 5d‐e). These results indicate obvious curative effects of MoS_2_ NF + US, and Sv‐MoS_2_ NF + US groups. Furthermore, the sonodynamic effects of MoS_2_ NF and Sv‐MoS_2_ NF were investigated by histopathological assessments. Hematoxylin and eosin (H&E) analysis shows karyorrhectic debris and significant karyolysis in the MoS_2_ NF + US and Sv‐MoS_2_ NF + US groups (Figure 5f). Tumors were further stained with DCFH‐DA and immunohistochemical terminal deoxynucleotidyl transferase‐mediated dUTP nick‐end labeling (TUNEL). Strong green fluorescence can be observed in the MoS_2_ NF + US and Sv‐MoS_2_ NF + US groups, which indicates the high ROS generation in the tumors after US irradiation. TUNEL analysis exhibits severe tumor apoptosis in the MoS_2_ NF + US and Sv‐MoS_2_ NF + US groups (Figure 5h). Those results show clearly that MoS_2_ NF and Sv‐MoS_2_ NF can act as effective sonosensitizers for SDT. What's more, the histopathological results also show that a higher capacity of Sv‐MoS_2_ NF as sonosensitizer than MoS_2_ NF.

### 2.6 Biosafety Evaluation of MoS_2_

**Figure 6:**
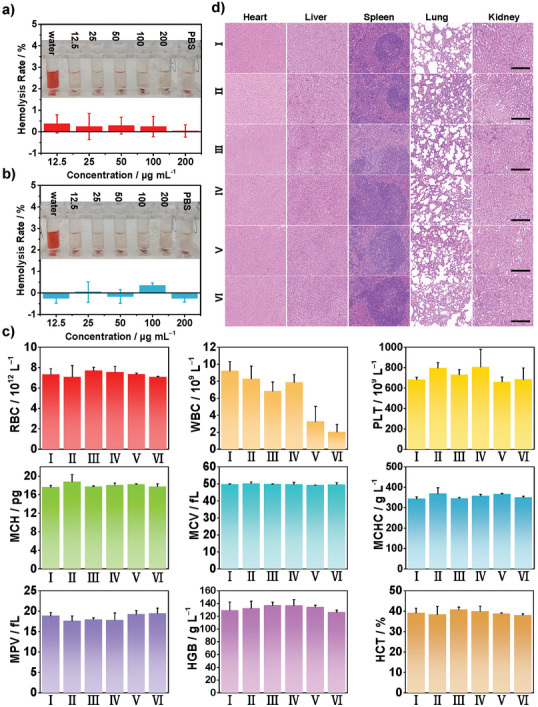
The biosafety of MoS_2_ NF and Sv‐MoS_2_ NF. The hemolysis rate of red blood cells (RBC) incubating with different concentrations of a) MoS_2_ NF and b) Sv‐MoS_2_ NF. c) Blood biochemistry and hematology analysis including white blood cells (WBC), RBC, hemoglobin (HGB), hematocrit (HCT), mean corpuscular volume (MCV), mean corpuscular hemoglobin (MCH), mean corpuscular hemoglobin concentration (MCHC), mean platelet volume (MPV), and platelets (PLT) of different groups (I = Control, II = MoS_2_ NF, III = Sv‐MoS_2_ NF, IV = US, V = MoS_2_ NF + US, VI = Sv‐MoS_2_ NF + US). Data are presented as mean ± SD (*n* = 3). d) H&E stain assay for major organs with different treatments. Scale bars = 200 µm.

The biosafety of MoS_2_ NF and Sv‐MoS_2_ NF was also investigated in vitro and in vivo. Different concentrations of MoS_2_ NF and Sv‐MoS_2_ NF were incubated with NIH‐3T3 cells, showing high biosafety from 0 to 200 µg mL^−1^ (Figure S20, Supporting Information)_._ The hemolysis assay was carried out to make sure that the materials could be injected intravenously. Both the hemolysis rates of MoS_2_ NF and Sv‐MoS_2_ NF are lower than 5%, revealing that they are safe for i.v. (**Figure** **6**a‐b). Furthermore, the biodistribution of Mo in main organs and tumor were detected by inductively coupled plasma mass spectrometry (ICP‐MS) to understand whether the materials were biodegraded after the therapeutic process. There is no significant difference among the groups in Figure S21 (Supporting Information). The blood biochemistry and hematology analysis of mice were measured for 18 days after *i.v*. of MoS_2_ NF and Sv‐MoS_2_ NF (Figure 6c). In addition, H&E analysis shows no pathological change or inflammatory response in the main organs of mice after an 18‐days therapeutic duration (Figure 6d). These results reveal that Sv‐MoS_2_ NF have great biocompatibility.

## 3 Conclusion 

Based on our findings, we have successfully developed Sv‐MoS_2_ NF with piezoelectric properties for SDT. Our research has shown that Sv‐MoS_2_ NF is capable of achieving increased piezoelectric polarization and band tilt under US through the construction of S‐vacancies, which enhances electron‐hole pairs separation and carriers migration, ultimately resulting in higher ROS production. Owing to the high SDT property of the Sv‐MoS_2_ NF originating from the rich vacancy, we have observed a remarkable tumor inhibition rate of 85.4%, alongside satisfactory biocompatibility. This work exhibits a novel piezo‐sonosensitizer, as well as provides a unique strategy for improving the performance of sonosensitizer, resulting in a new paradigm for the design of effective sonosensitizers and their application in therapy.

## Conflict of Interest

The authors declare no conflict of interest.

## Supporting information

Supporting Information

## Data Availability

The data that support the findings of this study are available from the corresponding author upon reasonable request.
