# Supplementary material for: Vacancy Augmented Piezo‐Sonosensitizer for Cancer Therapy
Source: Adv Sci (Weinh). 2023 Jul 3;10(26):2301152. doi: 10.1002/advs.202301152 (PMC10502820; doi:10.1002/advs.202301152)
Supplement: Supplementary file 1 — Supporting Information [file ADVS-10-2301152-s001.pdf]

ADVANCED SCIENCE
Open Access

Supporting Information

for *Adv. Sci.*, DOI 10.1002/advs.202301152

Vacancy Augmented Piezo-Sonosensitizer for Cancer Therapy

Qingyuan Wu, Jie Zhang, Xueting Pan, Zhijun Huang, Haoyuan Zhang, Juan Guo, Yun Xue, Rui Shi and Huiyu Liu**

Vacancy augmented piezo-sonosensitizer for cancer therapy

Qingyuan Wu, Jie Zhang, Xueting Pan, Zhijun Huang, Haoyuan Zhang, Juan Guo, Yun Xue, Rui Shi, Huiyu Liu**

Materials: Na_2SO_4 was obtained from Beijing Chemical Works. 9,10-diphenylanthracene (DPA) and methylene blue (MB) were obtained from Macklin. $(NH_4)_2MoS_4$ was obtained from HEOWNS Biochemical Technology Tianjin Co., Ltd. $N_2H_4 \cdot H_2O$ was purchased from Sinopharm Chemical Reagent Beijing Co. Ltd. 5,5-dimethyl-1-pyrroline N-oxide (DMPO) was obtained from Dojindo Molecular Technologies Shanghai Co. Ltd. Calcein acetoxymethyl ester (Calcein-AM), propidium iodide (PI), methyl thiazolyl tetrazolium (MTT) assay kit, 2',7'-dichlorodihydrofluorescein diacetate (DCFH-DA), dimethyl sulphoxide (DMSO), and Hematoxylin and eosin (H&E) were purchased from Beijing Solarbio Science &Technology (China). All reagents were used as received without any further purification.

Preparation of MoS_2 NF: The synthesis was carried out based on previous reports with slight modifications.[1] In the first step, 55 mg of $(NH_4)_2MoS_4$ was ground into powder using mortar. Then, 25 mL of deionized water mixed with the $(NH_4)_2MoS_4$ powder, was stirred at 300 rpm for 20 min. Subsequently, the rufous mixed solution was dissolved with an ultrasonic bath. After 10 min bath sonication, 250 μL of $N_2H_4 \cdot H_2O$ was dropped into the solution and the solution was treated with an ultrasonic bath for another 30 min. The temperature of an ultrasonic bath was kept below 30 °C during the whole process of bath sonication. Finally, the homogeneous

solution was transferred into a Teflon-lined stainless-steel autoclave and heated from room temperature to 200 °C for 12 h. The obtained MoS_2 NF were dispersed in deionized water and centrifuged at 10000 rpm for 7 min. After being washed three times, the MoS_2 NF were dried by a freeze dryer.

Defect engineering of MoS_2 NF: The MoS_2 NF (20 mg) were treated with H_2O_2 (100 μM, 20 mL) for 10, 30, 50, 70, and 90 min, respectively. After that, the solution was centrifuged at 10000 rpm for 7 min and dried by freeze dryer.

Characterization: The structure and component of MoS_2 were investigated by the powder X-ray diffraction analysis (Bruker D8 Advance), X-ray photoelectron spectroscopy (ESCALab220i-XL), ultraviolet-visible spectroscopy (Shimadzu-UV-2600). The morphology and size of MoS_2 were characterized by transmission electron microscopy (HITACHI HT-7700), high-resolution transmission electron microscopy, and energy-dispersive X-ray spectroscopy (JEM-3010). The zeta potential of MoS_2 was measured by dynamic laser scattering (Malvern Zetasizer Nano ZS). An inductively coupled plasma optical emission spectrometer (ICP-OES) was performed to measure the amount of molybdenum element. The signal of •OH was detected by an electron spin resonance spectrometer (ESR, Bruker EMXplus). The piezoelectric parameters of MoS_2 were detected by a piezoelectric atomic force microscope (MFP-3D Infinity). Confocal laser scanning microscopy (CLSM) images were shot with a Leica TCS SP8 confocal fluorescence microscope (Leica Microsystems). The photoacoustic images were obtained by the MSOT inVision 256-TF photoacoustic imaging system (iThera Medical, Germany).

Hydroxyl radical (•OH) generation experiments: •OH generation was evaluated by DMPO and the signals were detected by ESR spectrometer. 10 μL DMPO (180 mM, water solution) was mixed with 50 μL MoS_2 NF at 100 μg mL^{-1} and irradiated by US (1.0 MHz, 1.5 W cm^{-2}, 1 min). The UV–Vis method was also used for detecting •OH. MB (300 μg mL^{-1}, 50 μL) and MoS_2 NF (100 μg mL^{-1}, 2.5 mL) were mixed. Irradiating by the US (1.0 MHz, 1.5 W cm^{-2}) for different times, the absorbance changes of MB at 665 nm were used to quantify the decomposition rate.

•O_2^- generation experiments: •O_2^- generation was evaluated by DMPO and the signals were detected by ESR spectrometer. 10 μL DMPO (720 mM, DMSO solution) was mixed with 50 μL MoS_2 NF at 100 μg mL^{-1} and irradiated by the US (1.0 MHz, 1.5 W cm^{-2}, 3 min).

Electrochemical measurement: The electrochemical test was operated in a standard three-electrode configuration with carbon clot as the working electrode, carbon rod as the counter electrode, and Ag/AgCl/saturated KCl as the reference electrode. In the electrochemical impedance experiment and Mott-Schottky test, 0.1 M Na_2SO_4 (pH7) was used as the electrolyte. The electrode potential versus Ag/AgCl electrode was converted to a reversible hydrogen electrode (RHE) potential according to the Nernst equation: $V_{RHE} = V_E + 0.059 \times pH + V_{0\ Ag/AgCl}$, which $V_{0\ Ag/AgCl}$ = 0.224V in 25 ^oC, and V_E was the potential obtained against Ag/AgCl/saturated KCl reference electrode.

Piezopotential estimation: the piezopotentials (V_p) were estimated according to the equation:[2]

$$V_p = \frac{W_3 T_3 d_{33}}{\varepsilon_0 \varepsilon_r}$$

Here, W_3 is the thickness of the piezoelectric nanosheet, T_3 is the applied stress in the normal direction of the nanosheet, d_{33} is the piezoelectric constant, ε_0 is the vacuum permittivity, and ε_r is the relative permittivity in the normal direction. The W_3, T_3, and ε_r of the MoS_2 NF are estimated to be about 0.68 nm, 1×10^8 Pa, and 3.3, respectively. According to the piezoelectric response constants calculated on the amplitude-voltage butterfly loops, the piezoelectric potential of MoS_2 NF and Sv-MoS_2 NF can be estimated as 0.42 V and 0.69 V, respectively.

Photocatalytic experiment: The UV–Vis method was also used for detecting •OH generation under NIR light irradiation. MB (300 μg mL^{-1}, 50 μL) and MoS_2 NF (100 μg mL^{-1}, 2.5 mL) were mixed. Irradiating by NIR light (808 nm, 1.5 W cm^{-2}) for different times, the absorbance changes of MB at 665 nm were used to quantify the decomposition rate.

Cellular experiments: 4T1 breast cancer cells were cultivated in Dulbecco's modified Eagle's medium (DMEM, containing 10% fetal bovine serum, 1% of penicillin, and streptomycin) with 5% CO_2 at 37 °C.

MTT assay tests: To investigate the cytotoxicity of MoS_2 NF, 4T1 cells were seeded in a 96-well plate with a density of 10^4 cells per well. After 24 h incubation, MoS_2 NF with different concentrations (0, 6.25, 12.5, 25, 50, 100 μg mL^{-1}) was incubated with the cells for another 24 h. Then, the MTT assay was carried out according to a standard protocol to determine cell viability. To investigate the therapeutic effect of MoS_2 NF, 4T1 cells were seeded in a 96-well plate by 10^4 cells

per well. After 24 h incubation, MoS_2 NF with different concentrations (0, 12.5, 25, 50, 100 μg mL^{-1}) were incubated with the cells for another 12 h. Then, the cells were irradiated by the US (1.0 MHz, 1.5 W cm^{-2}, 2 min, 50% duty cycle) and incubated for 12 h. Finally, the MTT assay was used for detecting the cell viability.

CLSM measurements: The therapy effect of MoS_2 NF was also evaluated by CLSM. First, 2×10^5 cells were seeded on confocal dishes with a glass-bottom insert and incubated for 24 h. After that, the culture medium was removed. Subsequently, MoS_2 NF (100 μg mL^{-1}) of DMEM solution was added to the dishes and incubated for 12 h. The dishes were irradiated with or without US (1.0 MHz, 1.5 W cm^{-2}, 2 min, 50% duty cycle), respectively. After being cultivated for another 12 h, the cells were incubated with calcein-AM and PI co-staining (30 min, 37 °C) and then observed by CLSM. The ROS generation *in vitro* was detected by CLSM. Firstly, 2×10^5 cells were seeded on confocal dishes with a glass-bottom insert and incubated for 24 h. After that, the culture medium was removed. Subsequently, MoS_2 NF (100 μg mL^{-1}) of DMEM solution was added into the dishes and incubated for 12 h. The cells were incubated with DCFH-DA (25 min, 37 °C) and irradiated with or without US (1.0 MHz, 1.5 W cm^{-2}, 2 min, 50% duty cycle), respectively. Then the dishes were observed by CLSM.

Hemolysis Assay: The hematotoxicity of MoS_2 NF was assessed by using mice's red blood cells (RBCs). The blood of BALB/c mice was centrifugated and washed by PBS at 3000 rpm at 4 °C three times to remove white blood cells and plasma. MoS_2 NF (1 mL, 0.1 mg mL^{-1}) of PBS solution was mixed with 0.3 mL of freshly isolated

blood. Then, the mixture was incubated at 37 °C for 1 h. PBS and ultrapure water were represented as negative and positive controls, respectively. The mixtures were centrifuged. The supernatant absorbance at 540 nm was measured. The hemolysis ratio was calculated as hemolysis rate % = (sample absorbance − negative control absorbance) / (positive control absorbance − negative control absorbance) × 100%.

4T1 xenograft model establishment: Specific pathogen-free (SPF) BALB/c female mice (5 weeks) were obtained from Beijing Vital River Laboratory Animal Technology Co., Ltd. 25 μL of PBS with 4T1 cells (1×10^4) was mixed with 25 μL of matrigel. Then the mixture was injected subcutaneously to establish the animal tumor model.

***In vivo* PA Imaging:** MoS_2 NF (100 μL, 1 mg mL^{-1}) was tail intravenous injected into 4T1 tumor-bearing mice. PA images were captured by real-time multi-spectral optoacoustic tomography at corresponding time points postinjection (3, 6, 9, 12, 18, 24, 36, and 48 h).

***In vivo* sonodynamic therapy of MoS_2 NF:** BALB/c female mice (6 weeks) were purchased from Beijing Vital River Laboratory Animal Technology Co., Ltd. All animal experiments were approved by the local animal care committee. 4T1 tumor-bearing mice with tumor size of 60 mm^3 were divided into six groups (n = 5) at random: 1) control (only PBS injection); 2) US group (1.0 MHz, 2.5 W cm^{-2}, 50% duty cycle, 5 min); 3) MoS_2 NF group (tail intravenous injection of MoS_2 NF, 20 mg kg^{-1}); 4) Sv-MoS_2 NF group (tail intravenous injection of Sv-MoS_2 NF, 20 mg kg^{-1}); 5) MoS_2 NF + US group (tail intravenous injection of MoS_2 NF, 20 mg kg^{-1}, 1.0

MHz, 2.5 W cm^{-2}, 50% duty cycle, 5 min); and 6) Sv-MoS_2 NF + US group (tail intravenous injection of Sv-MoS_2 NF, 20 mg kg^{-1}, 1.0 MHz, 2.5 W cm^{-2}, 50% duty cycle, 5 min). Body weights and tumor sizes were measured and recorded every two days. The tumor volume = width^2 × length/2. On the 18th day, all the mice were sacrificed. All of the tumor and major organs were collected, sliced, and fixed for H&E staining. Tumor inhibition rate (%) = (1-V / V_0) × 100% (where V_0 and V are the tumor volume of the control group and the other groups on the 18th day, respectively).

Statistical Analysis: All the experiments were performed at least in triplicate. The statistical significance was determined using a two-tailed Student's test (*$P < 0.05$, **$P < 0.01$, and ***$P < 0.001$) unless otherwise stated.

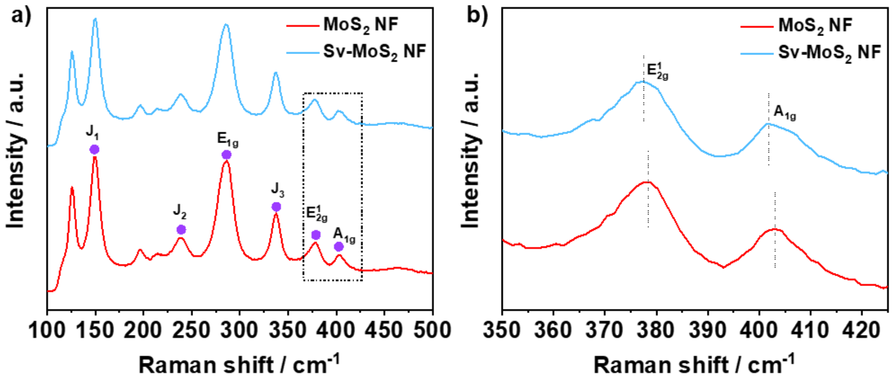

Figure S1. Raman spectra of MoS_2 NF and Sv-MoS_2 NF.

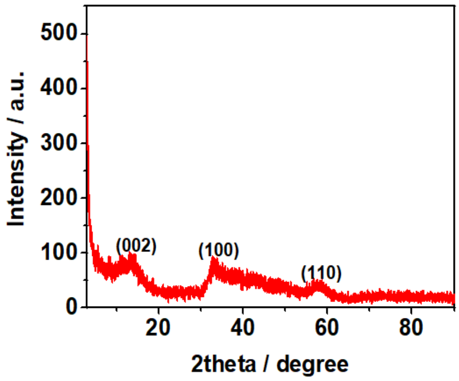

Figure S2. XRD pattern of MoS_2 NF.

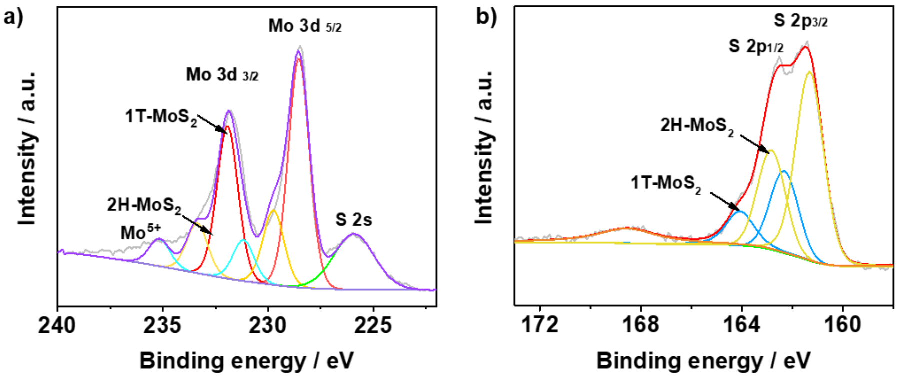

Figure S3. High-resolution a) Mo and b) S XPS spectra of MoS_2 NF.

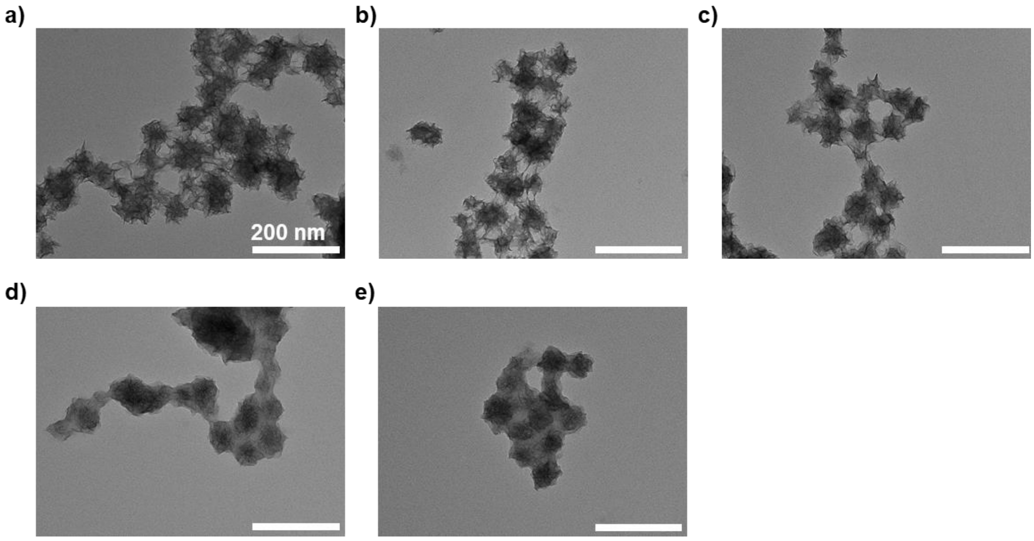

Figure S4. TEM images of a) M10, b) M30, c) Sv-MoS_2 NF, d) M70, e) M90.

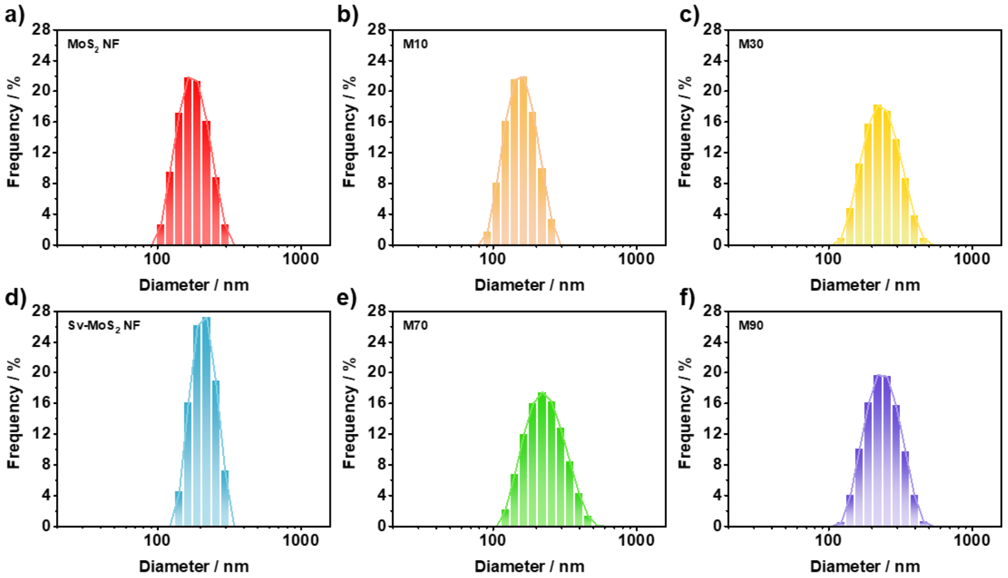

Figure S5. The DLS detected hydrated particulate sizes of a) MoS_2 NF, b) M10, c) M30, d) Sv-MoS_2 NF, e) M70, f) M90.

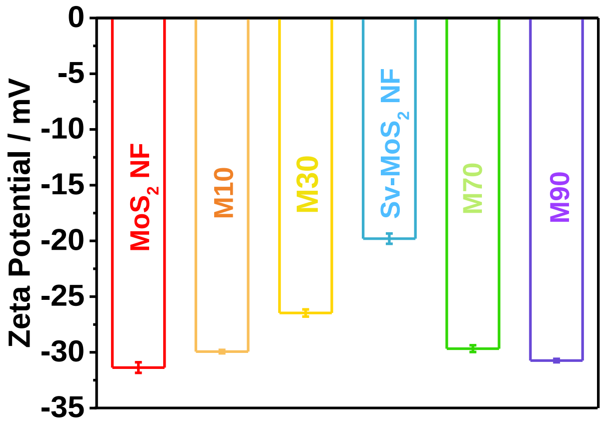

Figure S6. Zeta potentials of a) MoS_2 NF, b) M10, c) M30, d) Sv-MoS_2 NF, e) M70, and f) M90.

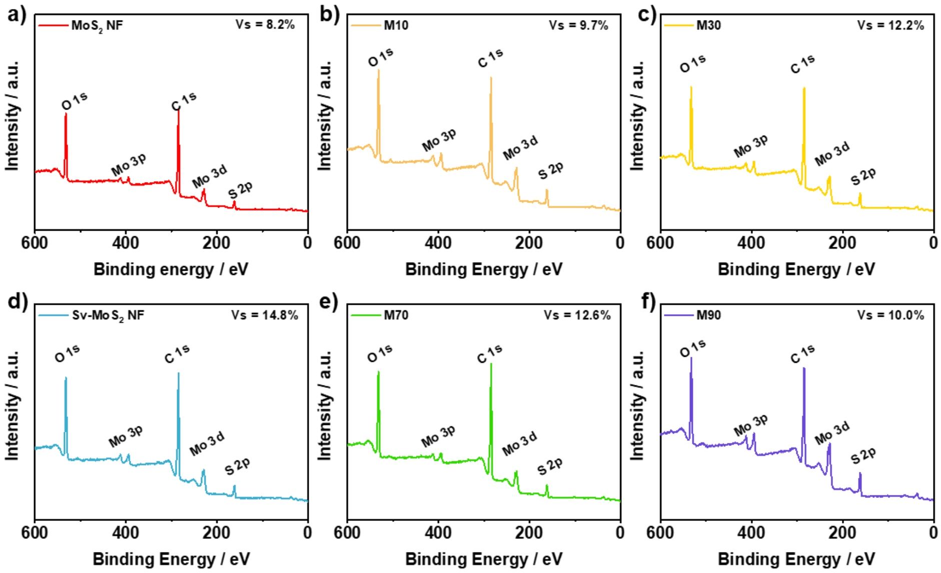

Figure S7. XPS spectra and the S-vacancy concentrations of a) MoS_2 NF, b) M10, c) M30, d) Sv-MoS_2 NF, e) M70, and f) M90.

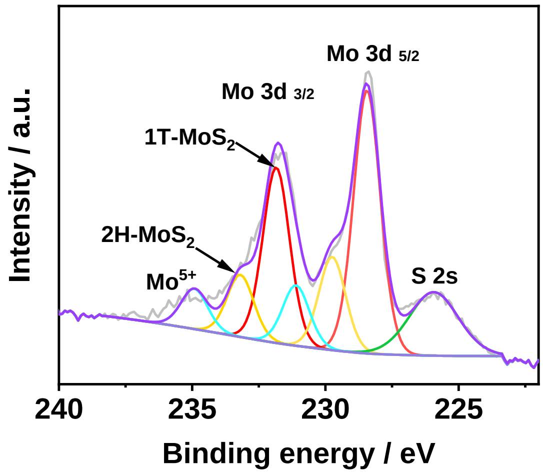

Figure S8. High-resolution XPS spectrum of Sv-MoS_2 NF.

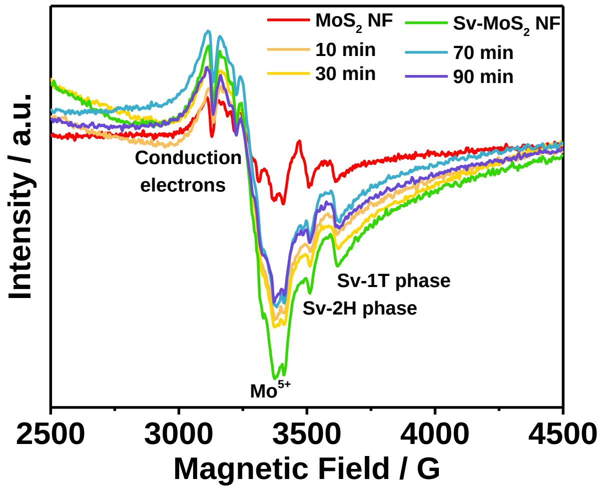

Figure S9. ESR spectra of MoS_2 NF, M10, M30, Sv-MoS_2 NF, M70, and M90.

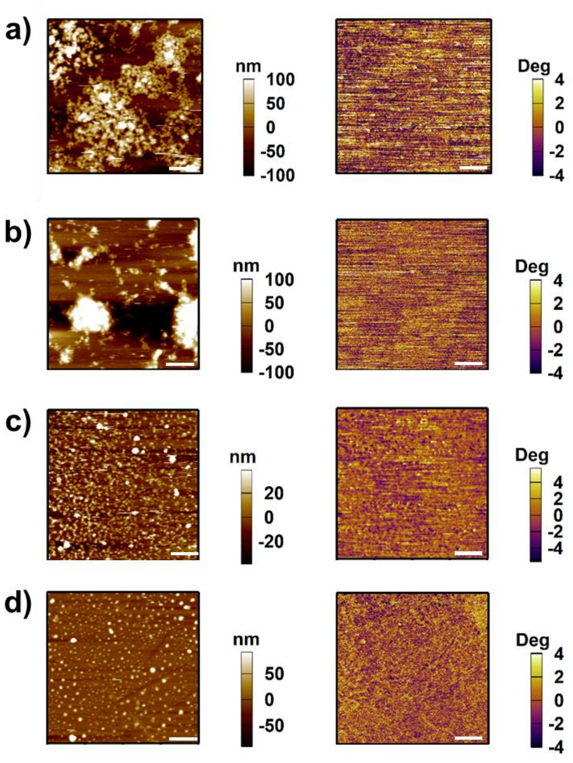

Figure S10. AFM and PFM phase images of a) M10, b) M30, c) M70, and d) M90. Scale bar = 5 μm.

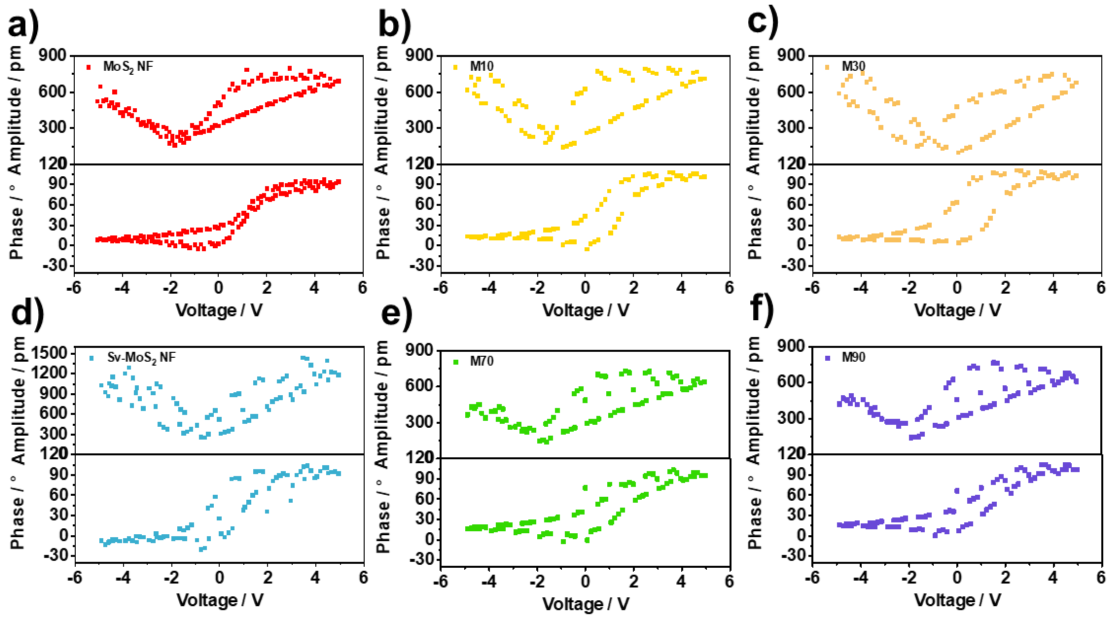

Figure S11. Piezoresponsive phase curves and amplitude curve of a) MoS_2 NF, b) M10, c) M30, d) Sv-MoS_2 NF, e) M70, and f) M90.

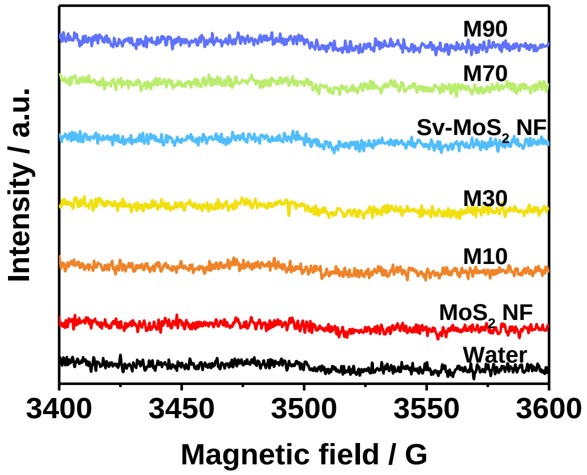

Figure S12. ESR spectra of •OH trapped by DMPO.

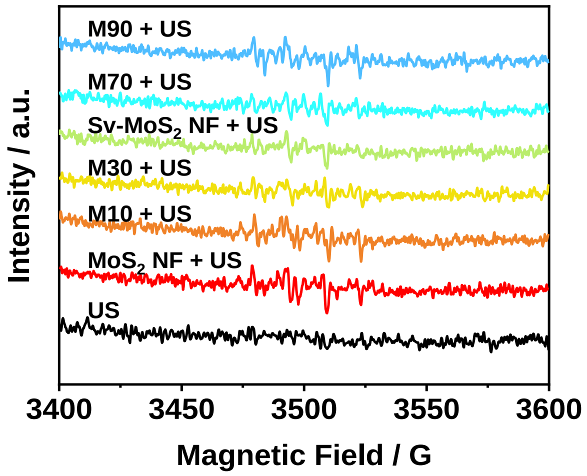

Figure S13. ESR spectra of $•O_2^-$ trapped by DMPO under US irradiation (3 min, 1.0 MHz, 1.5 W cm^{-2}).

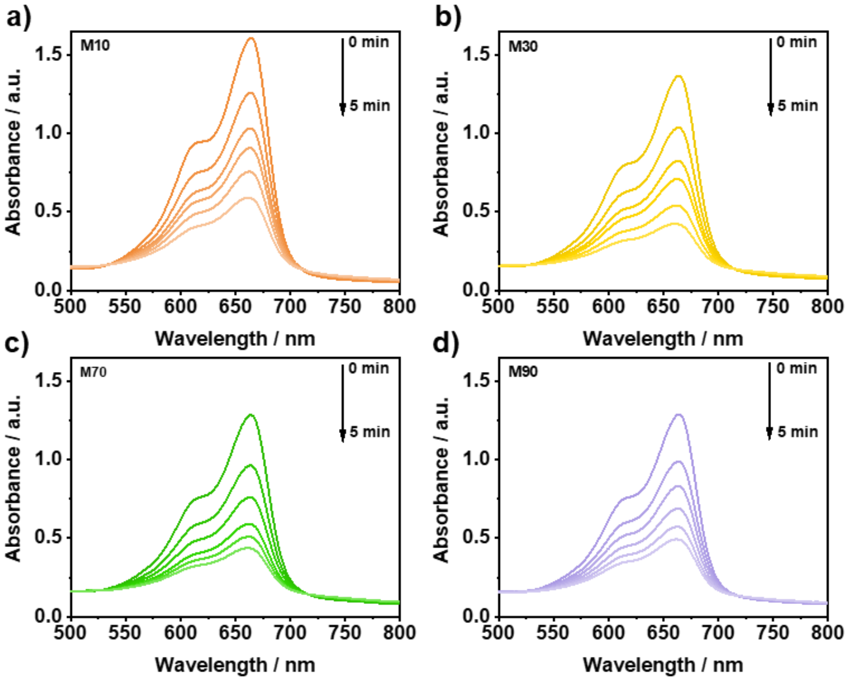

Figure S14. Time-dependent sono-degradation of MB indicating •OH generated by a) M10, b) M30, c) M70, and d) M90 under US irradiation (1.0 MHz, 1.5 W cm^{-2}).

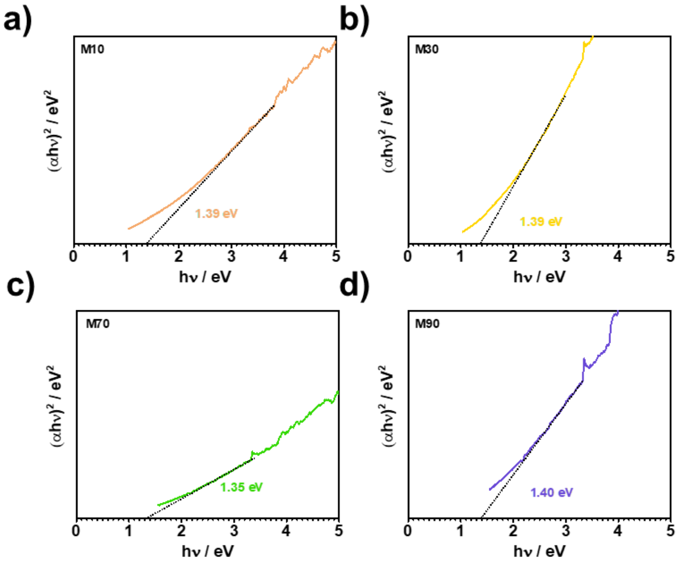

Figure S15. The optical band gaps of a) M10, b) M30, c) M70, and d) M90.

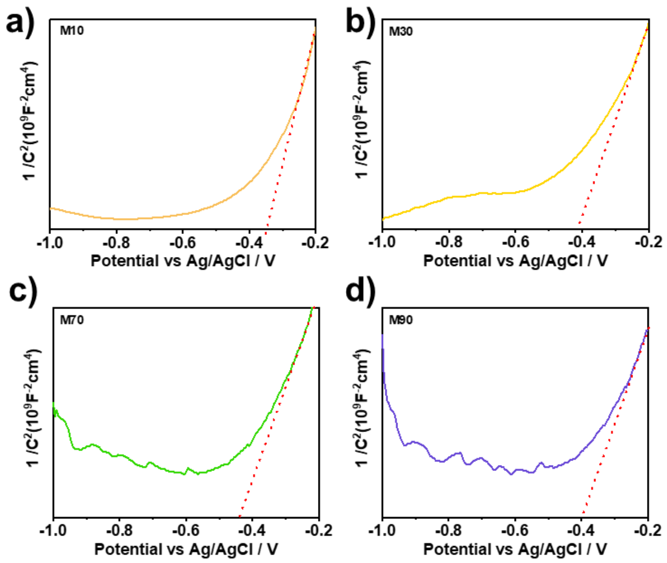

Figure S16. The Mott-Schottky plots of a) M10, b) M30, c) M70, and d) M90.

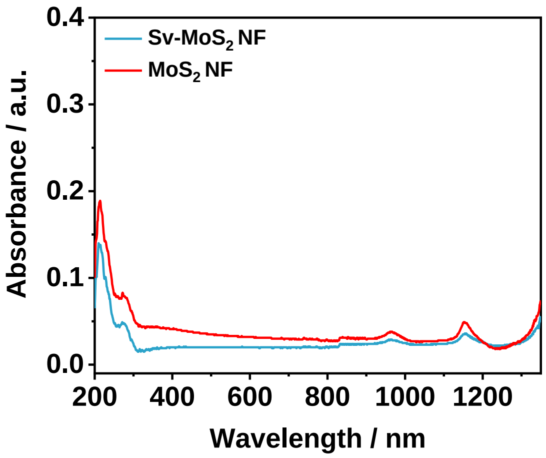

Figure S17. The UV–Vis-NIR spectra of MoS_2 NF and Sv-MoS_2 NF.

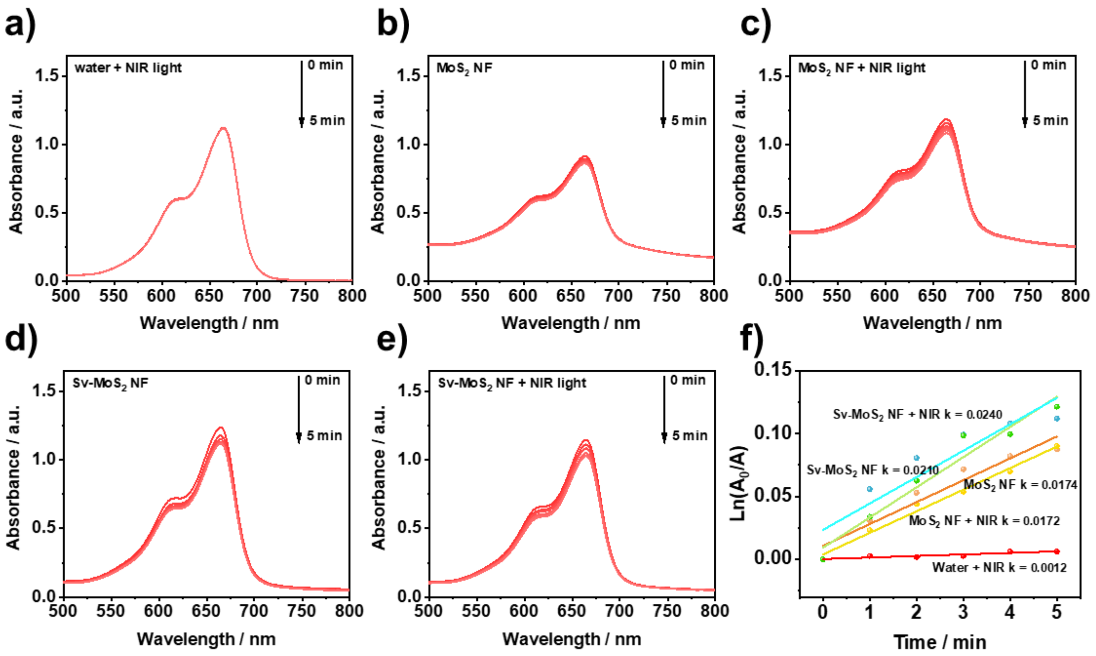

Figure S18. Photocatalytic MB decomposition by a) water + NIR light, b) MoS_2 NF, c) MoS_2 NF + NIR light, d) Sv-MoS_2 NF, and e) Sv-MoS_2 NF + NIR light. f) The calculated degradation rate constants. The parameters of NIR light were set as 1 W cm^{-2}.

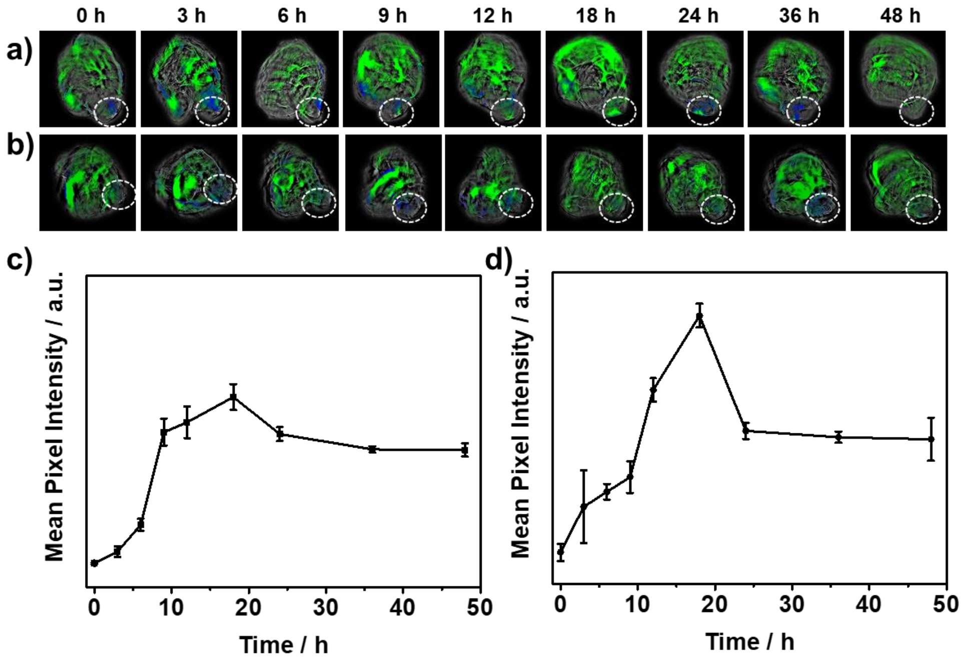

Figure S19. PA images of a) MoS_2 NF and b) Sv-MoS_2 NF after *i.v.* The mean PA signals of c) MoS_2 NF and d) Sv-MoS_2 NF.

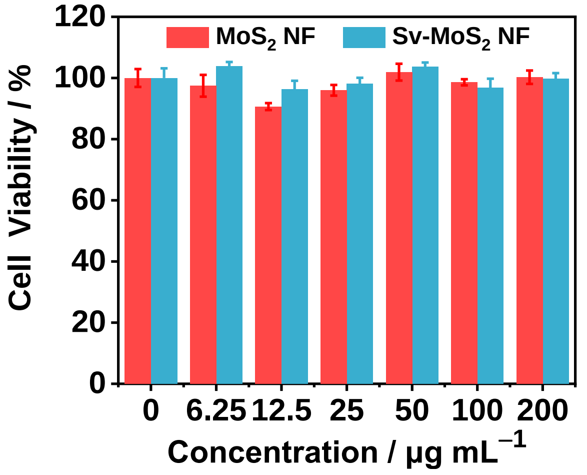

Figure S20. Relative cell viability of NIH-3T3 cells after being treated with MoS_2 NF and Sv-MoS_2 NF.

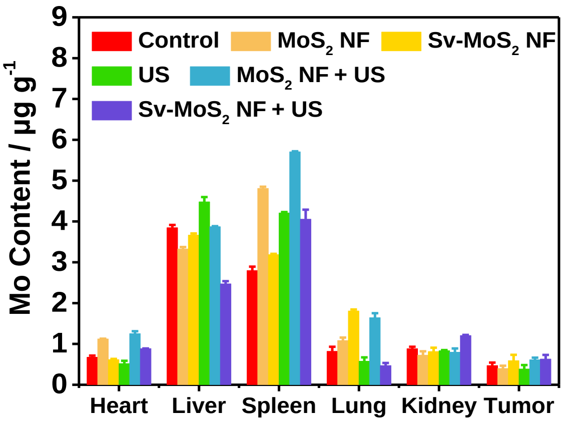

Figure S21. The biodistribution of Mo in major organs and tumor.

Table S1. The parameters of time-resolved transient photoluminescence spectra.

	τ_1 (ns)	τ_2 (ns)	B_1	B_2	τ_{ave} (ns)
MoS_2 NF	0.08595	2.778	1.181	0.001	0.158
Sv-MoS_2 NF	0.1016	1.764	1.115	0.004	0.199

τ_1: the fast decay time, τ_2: the slow decay time, τ_{ave}: the average decay time, B_1: the weighting factor of τ_1, B_2: the weighting factor of τ_2.

The fast decay time τ_1 of MoS_2 NF and Sv-MoS_2 NF are 0.08595 ns and 0.1016 ns, respectively. The slow decay time τ_2 are 2.778 ns and 1.76 ns. The average decay times are 0.082 ns and 0.108 ns of MoS_2 NF and Sv-MoS_2 NF, respectively.

[1]B. Liu, C. Li, G. Chen, B. Liu, X. Deng, Y. Wei, J. Xia, B. Xing, P. Ma, J. Lin, *Adv. Sci.* **2017**, 4, 1600540.

[2]H. You, Z. Wu, L. Zhang, Y. Ying, Y. Liu, L. Fei, X. Chen, Y. Jia, Y. Wang, F. Wang, S. Ju, J. Qiao, C. H. Lam, H. Huang, *Angew. Chem., Int. Ed.* **2019**, 58, 11779.
